# Methodology of emergency medical logistics for multiple epidemic areas in public health emergency

**DOI:** 10.1371/journal.pone.0253978

**Published:** 2021-07-26

**Authors:** Chunxia Hou, Huiyuan Jiang

**Affiliations:** School of Transportation and Logistics Engineering, Wuhan University of Technology, Wuhan, Hubei Province, P.R.China; Chongqing Jiaotong University, CHINA

## Abstract

Coronavirus disease 2019(COVID-19) has brought great disasters to humanity, and its influence continues to intensify. In response to the public health emergencies, prompt relief supplies are key to reduce the damage. This paper presents a method of emergency medical logistics to quick response to emergency epidemics. The methodology includes two recursive mechanisms: (1) the time-varying forecasting of medical relief demand according to a modified susceptible-exposed-infected- Asymptomatic- recovered (SEIAR) epidemic diffusion model, (2) the relief supplies distribution based on a multi-objective dynamic stochastic programming model. Specially, the distribution model addresses a hypothetical network of emergency medical logistics with considering emergency medical reserve centers (EMRCs), epidemic areas and e-commerce warehousing centers as the rescue points. Numerical studies are conducted. The results show that with the cooperation of different epidemic areas and e-commerce warehousing centers, the total cost is 6% lower than without considering cooperation of different epidemic areas, and 9.7% lower than without considering cooperation of e-commerce warehousing centers. Particularly, the total cost is 20% lower than without considering any cooperation. This study demonstrates the importance of cooperation in epidemic prevention, and provides the government with a new idea of emergency relief supplies dispatching, that the rescue efficiency can be improved by mutual rescue between epidemic areas in public health emergency.

## Introduction

Coronavirus disease 2019(COVID-19) has been an immense challenge for humanity. WHO has made the assessment that COVID-19 can be characterized as a pandemic, while the pandemic will eventually recede, but there can be no going back to business as usual [1, 2]. As well as COVID-19, public health emergencies always result in health threats, economic losses, psychological suffering and so no [3].

In response to public health emergencies, prompt relief supplies are key to reduce the loss of life and other influence. Quick response to the urgent need of supplies in affected areas is a critical issue for emergency logistics, which has aroused growing concerns and research interests recently due to the occurrence of several infectious disease (e.g., COVID-19, H1N1 influenza, Ebola virus, SARS, and so on).

Emergency logistics mainly involves the location of emergency logistics facilities, transportation supplies of emergency materials, the transport vehicle routing optimization, and the combination of above problems. Some literature focus on transportation supplies of emergency materials [4–10], and some on the other issues [11–18]. This paper focuses on transportation supplies of emergency materials. In the distribution of emergency supplies, the demand forecast of relief supplies is a critical issue, which is related to the number of affected people. When a natural disaster occurs, the affected areas and the number of affected people is basically fixed, which determined by the disaster magnitude. Zhan and Liu (2013), Ge and Liu (2014) proposed a stochastic programming model for relief supplies allocation based on complex disaster scenarios, respectively [5, 14]. Mete and Zabinsky (2010) presented a forecasting and stochastic optimization approach for the storage and distribution problem of medical supplies under a wide variety of possible disaster types and magnitudes [19]. The disaster level can be estimated according to the historical disaster data. Sheu (2007) presented a three-layer emergency logistics framework, and a time-varying relief demand forecast model, in which the number of affected people was assumed to follow a Poisson process [8]. In some literatures, the uncertain information of demand is treated by fuzzy number. Wang, et al. (2012) proposed a multi-objective nonlinear integer programming model with the objectives of total transportation time and cost, in which the demand was processed by fuzzy function [20].

Different from general emergency logistics, emergency medical logistics is unique in three aspects that increase the complexity and difficulty in solving the relief logistics problems, particularly in terms of emergency logistics distribution. First, compared with natural disasters, the spread of infectious disease is a time-continuing process, so the distribution of emergency medical supplies must be multi-cycle. Second, infectious disease can be transmitted rapidly from one region to another, the number of affected areas will change with the transmission of epidemic. In addition, due to the different medical conditions in different regions, the critical disease parameters such as transmission rate varies in different regions. It means that the number of demand point and the quantity of demanded supplies are varying in different rescue period. Third, the demand-related information changes throughout the public health emergency, such as the infectious diseases transmission, infection, recovery, and mortality rates. Additionally, the information is hysteretic due to the incubation period of the infectious diseases [16]. According to the difference, epidemic diffusion rule plays a critical role in demand forecast of public health emergencies. Some literature on emergency medical logistics proposed a two-stage model including a modified epidemic susceptible-exposed-infected-recovered (SEIR) model and a linear programming model, in which SEIR model is developed to forecast time-varying demand [7, 15–17, 21]. As shown in the above literature, Epidemic diffusion model is critical in emergency medical logistics problems. Most epidemic diffusion models, including susceptible-infectious (SI), susceptible-infectious-removed-susceptible (SIRS), susceptible-infectious-susceptible (SIS), susceptible-infectious-removed (SIR), susceptible-exposed-infectious (SEI), and SEIR, are suitable for studying the general laws of epidemics [22]. Particularly, SEIR model has drawn considerable attention, which considers incubation period [7, 16, 17]. However, some of the COVID-19 infectors are asymptomatic, which did not be considered in SEIR models. Arino, et al. (2008) proposed the susceptible-exposed-infected- Asymptomatic- recovered (SEIAR) model to describe the asymptomatic patients [23]. In addition, infection shows migration and dispersal patterns, but most previous works have oversimplified the problem by ignoring the spread of disease between different regions [17]. Therefore, considering population mobility between different epidemic areas and the asymptomatic infectors, this work uses a modified SEIAR model to study the epidemic law.

Emergency logistics network generally includes relief suppliers, relief distribution centers, and affected areas [8]. Some literature focuses on the supply chain [6, 7, 24], some on the distribution chain [4, 5, 9, 18], and some on the whole logistics network [8, 14, 15, 25, 26]. This paper focuses on the relief supply chain. In the existing literature, emergency supplies are supplied only by the national Emergency Medical Reserve Center (EMRC), and the suppliers remain unchanged in multi-period [5, 7, 8, 11–16, 18]. Few studies considered various types of suppliers in the emergency logistics network [25]. The outbreak of infectious diseases is unpredictable, the distance between national EMRC and epidemic area may be faraway, so the timeliness and economy of the distribution of emergency medical reliefs cannot be guaranteed. Therefore, this study considers a variety of relief suppliers, including the national EMRCs, the less affected area of all epidemic areas, and e-commerce warehousing centers.

In the previous literature, multi-objective programming model and stochastic programming model are presented to optimize the medical relief supplies distribution, with different object functions according to varying concerns. Some studies proposed to minimize the distribution time and logistics cost, focusing on the timeliness and economy [4, 7]. Other objective functions include minimize mortality [27], the number of rescue point [28], the physical fragility and psychological fragility [16], the total number of infected individuals and deaths [17], and maximize level of satisfaction of the relief demand [8]. This paper establishes a multi-objective and multi-cycle dynamic stochastic optimization model to minimize transportation cost and time. The solution method mostly is heuristic algorithm, including genetic algorithm [12, 29], immune algorithm [10, 30], simulated annealing algorithm and so on. Based on the limited information and fuzzy numbers in public health emergency, some literatures developed fuzzy mathematical model to solve the distribution problem [6, 20, 26, 28]. Considering The distribution problem is a multi-objective stochastic programming problem with multiple rescue points, multiple demand points and a variety of relief materials, this paper uses a two-dimensional immune algorithm to solve the optimization model.

Particularly, the main contributions of this work are summarized below.

1. This study designs an emergency medical logistics network, in which the suppliers (rescue points) and the demanders (demand points) changes in every period. In addition, considering the mutual rescue between epidemic areas, the increasingly mature development of e-commerce, we take the national EMRCs, the less affected area of all nearby epidemic areas and the e-commerce warehousing centers as the relief suppliers.
2. This paper develops a modified SEIAR dynamically model, considering the population mobility between different areas, which can more accurately describe and predict the spread process of COVID-19. Additionally, we prove the superiority of the model through data comparison.
3. This work establishes a multi-period, multi-objective dynamic stochastic programming model, with multiple rescue points, multiple demand points and a variety of relief materials, and use the two-dimensional immune algorithm to solve the model. We conduct a case study using the real data of Hubei province, China, and a continuation study with experimental data to demonstrate the applicability of the proposed model.

The remainder of the paper is organized as follows. we present the methodological framework of the proposed approach, and develop the models in *Materials and methods*. We describe the algorithm in *Solution algorithm*, use numerical simulations to verify the effectiveness and advantages of the developed approach in *Numerical study and Discussion*, and draw the conclusions and recommendations on future studies in *Conclusions*.

## Materials and methods

This section presents the recommended Methodology. A modified SEIAR model is developed based on an emergency medical relief system specification to forecast time-varying demand. A multi-objective dynamic stochastic programming model raised for medical supplies distribution.

### System specification

**Fig 1:**
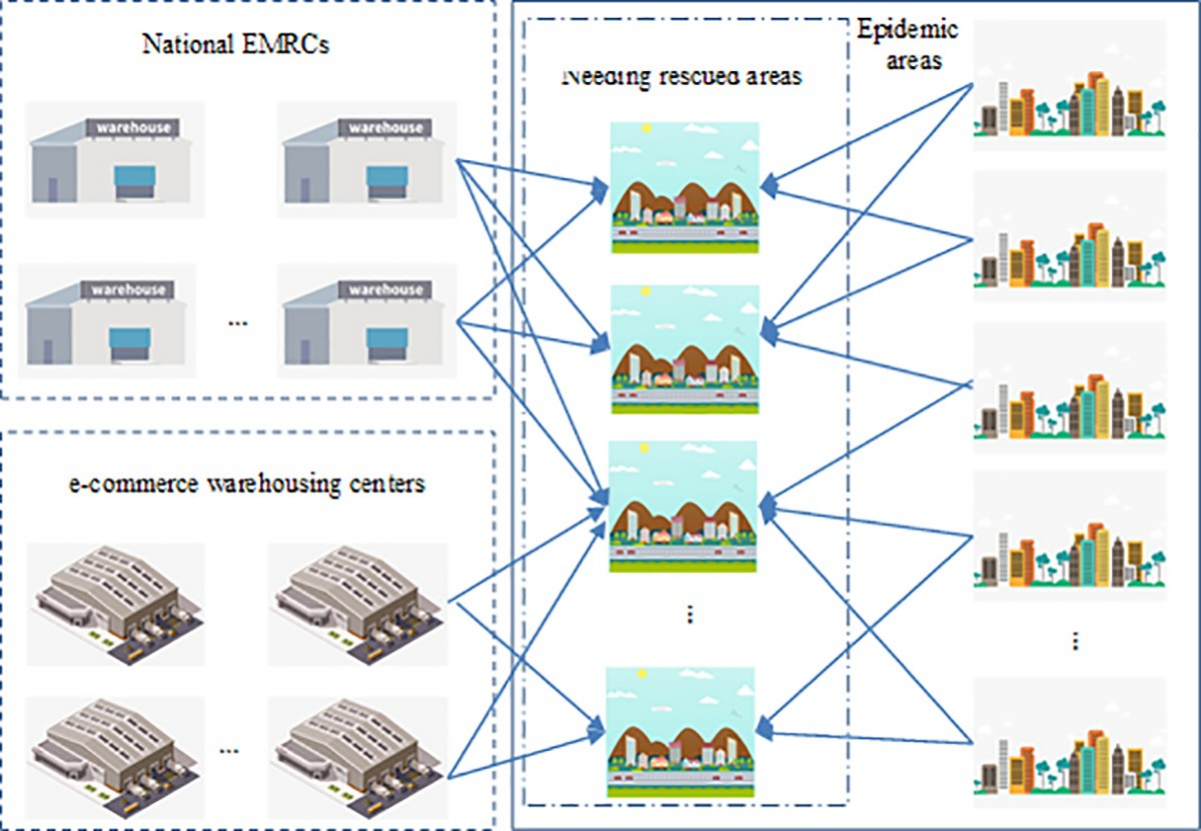
Conceptual framework of the specified emergency medical logistics network.

The hypothetical emergency medical logistics network considered in this study is depicted in Fig 1, which is a specific two-layer supply chain, involving two primary chain members: (1) rescue points, including the national EMRCs, epidemic areas which do not need to be rescued, and e-commerce warehousing centers. (2) demand points, the epidemic areas which need to be rescued. Here, relief suppliers refer to the private or public organizations, the national EMRCs are dominated by the national government, the epidemic areas’ EMRCs are possessed by the local government, and the e-commerce warehousing centers are owned to the private e-commerce enterprises.

When a new infectious disease outbreaks in a city, it will be quickly spread to the surrounding areas, due to the population migration. The government decides whether to issue an emergency medical response after the infectious disease outbreak, as well as which epidemic areas to rescue immediately, according to the transmission of the infectious disease.

First, EMRCs initiate a mechanism to forecast the duration of epidemic and time-varying medical relief demand by collecting and estimating epidemic parameter. Second, in order to determine the rescue epidemic areas, EMRCs need to assess whether the stored medical supplies can meet the demand in each epidemic area. Then, the mechanism of medical relief distribution is then triggered based on updated information.

**Fig 2:**
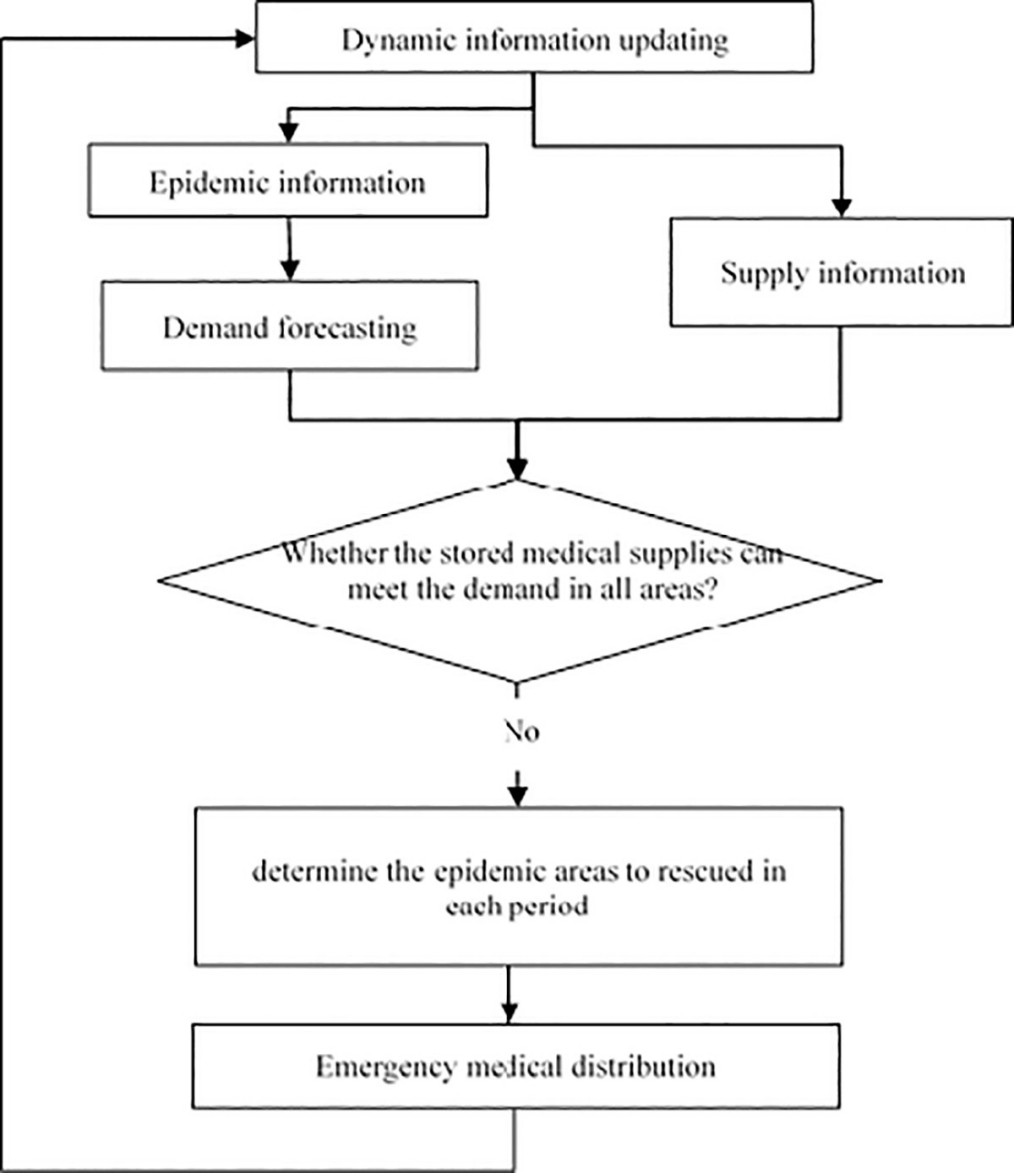
Sequence of operational procedures in emergency medical logistics.

Fig 2 presents the recurrent calculation step in emergency medical logistics. Supply and epidemic information are updated at the beginning of each step. The termination of rescue depends on whether the number of infected people in all epidemic areas is equivalent to zero.

Seven basic assumptions are made to facilitate model formulation:

1. The geographic information of epidemic areas is available, the location of national EMRCs and e-commerce warehousing centers are known because they have been established before emergencies.
2. The new infected areas are all around the initial epidemic area.
3. Emergency medical suppliers are known. The number and the type of available medical suppliers are identified at the beginning of each period.
4. Different medical reliefs cannot be loaded on a vehicle. Correspondingly, a vehicle is not allowed to load multiple medical reliefs.
5. All the medical reliefs needed in the epidemic areas are delivered once.
6. Differences in Transmission, infection, recovery, and mortality rates between different people are not considered.
7. Recovered individuals acquire permanent immunity.

Based on these assumptions, the next sections present the demand forecasting model and the distribution model, respectively.

### Model notations

Model notations that will be used throughout the rest of this paper are shown below:

Sets and indices:

*B*1      Set of all epidemic areas.

*B*2      Set of epidemic areas that need to be rescued.

*A*    Set of the national EMRCs.

*E*    Set of e-commerce warehousing centers.

*M*    Set of emergency medical supplies, *M* = (*m*1,*m*2,*m*3), *m*1, *m*2 and *m*3 are prophylactic, testing, and treatment medical supplies, respectively.

*N*_*j*_      Number of permanent people in epidemic area *j*.

*b*_*k*_      Epidemic area *k*, *b*_*k*_∈*B*1.

*b*_*j*_      Epidemic area *j* that need to be rescued, *b*_*j*_∈*B*2.

a_*i*_      National EMRC *i*, a_*i*_∈*A*.

*e*_*i*_      E-commerce warehousing centers *i*, *e*_*i*_∈*E*.

Transition parameters used to describe the rate of movement between different classes:

*λ*_*j*_      Transmission rate of epidemic area *j*.

*σ*_*j*_      Infection rate of epidemic area *j*.

*β*_*j*_      Asymptom rate of epidemic area *j*.

*γ*_*j*_      Recovery rate of epidemic area *j*.

*α*_*j*_      Disease mortality rate of epidemic area *j*.

*d*_*j*_      Natural mortality rate of epidemic area *j*.

Other parameters:

*a*^*m*^      Number of medical supplies *m* required by each demander per unit of time.

*δ*_*j*_(*t*)     Number of relief demanders. The number of people who need the prophylactic relief *m*1 is equal to *E*_*j*_(*t*)+I_*j*_(*t*)+*A*_*j*_(*t*), the number of people who need the testing relief *m*2 is equal to *E*_*j*_(*t*), and the number of people who need the treatment relief *m*3 is equal to *I*_*j*_(*t*).

*L*      Upper bound of the lead time, in order to ensure enough time available for the vehicles to return to the storage places and prepare for the next distribution.

*V*_*m*_      Available transportation capacity of vehicle to load relief supply *m*.

*g*      Unit transport time of vehicle.

*h*      Unit transport cost of vehicle.

$qa_{i}^{m}(t)$ Available amount of relief *m* in EMRC *a*_*i*_ in time period *t*.

$qb_{j}^{m}(t)$ Available amount of relief *m* in epidemic area *b*_*j*_ in time period *t*.

$qb_{k}^{m}(t)$ Available amount of relief *m* in epidemic area *b*_*k*_ in time period *t*.

$qe_{i}^{m}(t)$ Available amount of relief *m* in e-commerce warehousing center *e*_*i*_ in time period *t*.

$l_{a_{i}↔b_{j}}$ Distance between EMRC *a*_*i*_ and epidemic area *b*_*j*_.

$l_{b_{k}↔b_{j}}$ Distance between epidemic area *b*_*k*_ and *b*_*j*_.

$l_{e_{i}↔b_{j}}$ Distance between e-commerce warehousing center *e*_*i*_ and epidemic area *b*_*j*_.

*na*_*i*_      Available amount of vehicle in EMRC *a*_*i*_.

*nb*_*i*_      Available amount of vehicle in epidemic area *b*_*j*_.

*nb*_*k*_      Available amount of vehicle in epidemic area *b*_*k*_.

*ne*_*i*_      Available amount of vehicle in e-commerce warehousing center *e*_*i*_.

*T*_*ω*_      Transportation time threshold, penalty will be given if the transportation time exceeding *T*_*ω*_.

*ω*      Confidence level of meeting the emergency materials demand.

*β*      Penalty coefficient, the amount of increased objective function value due to per unit unsatisfied relief.

State variables:

*S*_*j*_(*t*)      Numbers of susceptible people in area *b*_*j*_ at moment *t*.

*E*_*j*_(*t*)      Numbers of exposed people in area *b*_*j*_ at moment *t*.

*I*_*j*_(*t*)      Numbers of infectious people in area *b*_*j*_ at moment *t*.

*A*_*j*_(*t*)      Numbers of asymptomatic people in area *b*_*j*_ at moment *t*.

*R*_*j*_(*t*)      Numbers of recovered people in area *b*_*j*_ at moment *t*.

$D_{j}^{m}(t)$ Demand for medical relief *m* in area *b*_*j*_ in time period *t*.

Decision variables:

$x_{ai}^{m}(t)$ Whether or not the EMRC *a*_*i*_ is chosen to send relief *m* to area *b*_*j*_ in time period *t*, $x_{ai}^{m}(t)\in \left\{0,1\right\}$, with 0 indicates it is not be chosen, and 1 means it is be chosen.

$y_{kj}^{m}(t)$ Whether or not the area *b*_*k*_ is chosen to send relief *m* to area *b*_*j*_ in time period *t*, $y_{kj}^{m}(t)\in \left\{0,1\right\}$, with 0 indicates it is not be chosen, and 1 meanes it is be chosen.

$z_{ej}^{m}(t)$ Whether or not the e-commerce warehousing center *e*_*i*_ is chosen to send relief *m* to area *b*_*j*_ in time period *t*, $z_{ej}^{m}(t)\in \left\{0,1\right\}$, with 0 indicates it is not be chosen, and 1 means it is be chosen.

$w_{aj}^{m}(t)$ The number of vehicles that be used to transport relief *m* from EMRC *a*_*i*_ to area *b*_*j*_ in time period *t*, which is an integer variable.

$w_{kj}^{m}(t)$ The number of vehicles that be used to transport relief *m* from area *b*_*k*_ to area *b*_*j*_ in time period *t*, which is an integer variable.

$w_{ej}^{m}(t)$ The number of vehicles that be used to transport relief *m* from e-commerce warehousing center *e*_*i*_ to area *b*_*j*_ in time period *t*, which is an integer variable.

### Relief demand forecasting model

This system forecasts the time-varying emergency medical demand of each affected area based on epidemic diffusion rules. Considering that there are asymptomatic infectious patients in some infectious diseases, this paper chooses the SEIAR model to describe the epidemic diffusion rules.

**Fig 3:**
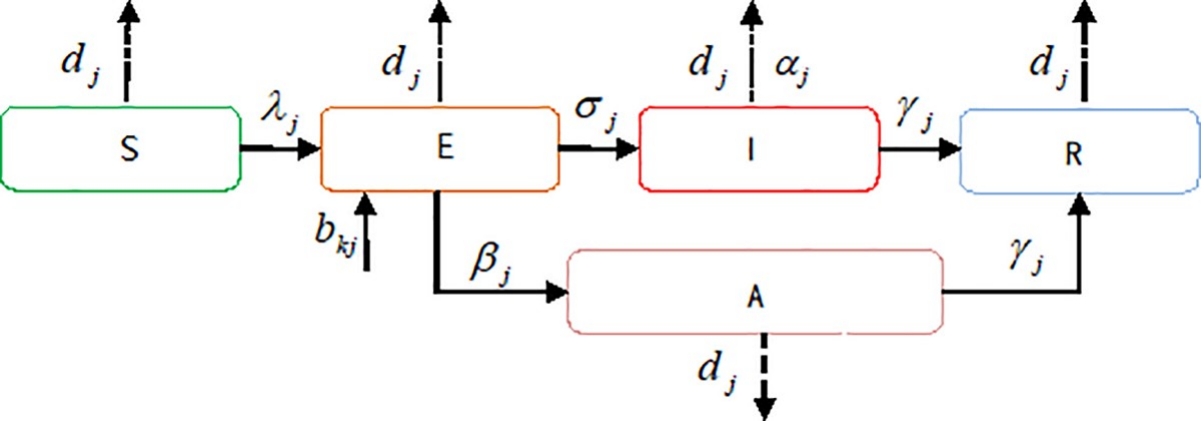
SEIAR model.

SEIAR model divided the people into five classes: S (susceptible), E (exposed), I (infectious), A (asymptomatic), and R (recovered). Fig 3 shows the relationship and transition among different groups in a specific area.

The model describes the transition among these classes with differential equations, including Eqs (1)–(5).


$$S_{j}(t+1)=S_{j}(t)-\lambda_{j}\left[I_{j}(t)+A_{j}(t)\right]\times \frac{S_{j}(t)}{N_{j}}-d_{j}S_{j}(t)$$ (1)

$$E_{j}(t+1)=E_{j}(t)+\lambda_{j}\left[I_{j}(t)+A_{j}(t)\right]\times \frac{S_{j}(t)}{N_{j}}-\sigma_{j}E_{j}(t)-\beta_{j}E_{j}(t)-d_{j}E_{j}(t)$$ (2)

$$I_{j}(t+1)=I_{j}(t)+\sigma_{j}E_{j}(t)-\gamma_{j}I_{j}(t)-(d_{j}+\alpha_{j})I_{j}(t)$$ (3)

$$A_{j}(t+1)=A_{j}(t)+\beta_{j}E_{j}(t)-\gamma_{j}A_{j}(t)-d_{j}A_{j}(t)$$ (4)

$$R_{j}(t+1)=R_{j}(t)+\gamma_{j}I_{j}(t)+\gamma_{j}A_{j}(t)-d_{j}R_{j}(t)$$ (5)

Eq (1) describes how the number of susceptible people varies in epidemic area *j*. This number decreases as susceptible people change to exposed, as well as die naturally. Eq (2) indicates the transition from susceptible to exposed groups and from exposed to infectious or asymptomatic groups, with natural deaths. Eq (3) describes the transition from exposed to infectious groups and from infectious to recovered groups, as well as natural and diseased deaths. Eq (4) indicates the transition from exposed to asymptomatic groups and from asymptomatic to recovered groups, with natural deaths. Eq (5) indicates how the number of recovered people varies.

Considering the cross-regional transmission of the infectious diseases, we propose a matrix B as migration of different epidemic areas, $B=\left[\begin{array}{lllll} b_{11} & \cdots & b_{1j} & \cdots & b_{1J}\\ \vdots & \ddots & & & \vdots \\ b_{j1} & & b_{jj} & & b_{jJ}\\ \vdots & & & \ddots & \vdots \\ b_{J1} & \cdots & b_{Jj} & \cdots & b_{JJ} \end{array}\right]$, the parameter *b*_*jk*_ is the mobility rate at which the population in *j* moves to *k*, *b*_11_ = *b*_22_ = ⋯ = *b*_*JJ*_ = 0, *b*_*jk*_≥0 and $\sum_{k=1}^{J}b_{jk}\leq 1$. Based on the migration matrix, this work constructs a modified SEIAR model, including Eqs (6)–(10). In this model, the population of each class is increased by immigrants moving in and decreased by immigrants moving out.


$$S_{j}(t+1)=S_{j}(t)-\lambda_{j}\left[I_{j}(t)+A_{j}(t)\right]\times \frac{S_{j}(t)}{N_{j}}-d_{j}S_{j}(t)+\sum_{k=1}^{J}b_{kj}S_{k}(t)-\sum_{k=1}^{J}b_{jk}S_{j}(t)$$ (6)

$$E_{j}(t+1)=E_{j}(t)+\lambda_{j}\left[I_{j}(t)+A_{j}(t)\right]\times \frac{S_{j}(t)}{N_{j}}-\sigma_{j}E_{j}(t)-\beta_{j}E_{j}(t)-d_{j}E_{j}(t)+\sum_{k=1}^{J}b_{kj}E_{k}(t)-\sum_{k=1}^{J}b_{jk}E_{j}(t)$$ (7)

$$I_{j}(t+1)=I_{j}(t)+\sigma_{j}E_{j}(t)-\gamma_{j}I_{j}(t)-(d_{j}+\alpha_{j})I_{j}(t)+\sum_{k=1}^{J}b_{kj}I_{k}(t)-\sum_{k=1}^{J}b_{jk}I_{j}(t)$$ (8)

$$A_{j}(t+1)=A_{j}(t)+\beta_{j}E_{j}(t)-\gamma_{j}A_{j}(t)-d_{j}A_{j}(t)+\sum_{k=1}^{J}b_{kj}A_{k}(t)-\sum_{k=1}^{J}b_{jk}A_{j}(t)$$ (9)

$$R_{j}(t+1)=R_{j}(t)+\gamma_{j}I_{j}(t)+\gamma_{j}A_{j}(t)-d_{j}R_{j}(t)+\sum_{k=1}^{J}b_{kj}R_{k}(t)-\sum_{k=1}^{J}b_{jk}R_{j}(t)$$ (10)

Through the modified SEIAR model, the number of exposed people and infectious people in each epidemic area at each period *t* can be obtained, and the time-varying forecast model [8] is established as follow.


$$D_{j}^{m}(t)=a^{m}\times \delta_{j}(t)\times L+z_{1-\alpha }\times STD_{j}^{m}(t)\times \sqrt{L}$$ (11)

Where $STD_{j}^{m}(t)$ is the standard deviation of demand for medical relief *m* in area *b*_*j*_ in time period *t*, the formula is as follows:

$$STD_{j}^{m}(t)=\frac{\sqrt{\sum_{t=0}^{N}{\left[D_{j}^{m}(t)-\bar{D_{j}^{m}(t)}\right]}^{2}}}{N-1}$$ (12)

In this study, three types of emergency medical supplies are considered: prophylactic supplies for susceptible people to reduce the infection rate (denoted as *m*1), testing supplies for explored people to lower the infection rate (denoted as *m*2), and treatment supplies for infectious people to lower the mortality rate (denoted as *m*3). Demand for prophylactic reliefs is correlated with the numbers of exposed, infectious, and asymptomatic people. Demand for testing reliefs is strongly correlated with the number of explored individuals. Demand for treatment reliefs is strongly correlated with the number of infectious individuals.

### Distribution decision model

In this section, a multi-objective stochastic programming model is formulated and applied to distribute urgent medical reliefs from multiple relief suppliers, including the national EMRCs, epidemic areas that do not need to be rescued, and e-commerce warehousing centers, to multiple epidemic areas that need to be rescued.

The objective functions are:

$$\min TC_{1}(t)=\underset{\begin{array}{l} a\in A,k\in B1,j\in B2,\\ e\in E,m\in M \end{array}}{\max}\left\{\begin{array}{l} x_{aj}^{m}(t)\times w_{aj}^{m}(t)\times l_{a_{i}↔b_{j}}\times g,y_{kj}^{m}(t)\times w_{kj}^{m}(t)\times l_{b_{k}↔b_{j}}\times g,\\ z_{ej}^{m}(t)\times w_{ej}^{m}(t)\times l_{e_{i}↔b_{j}}\times g \end{array}\right\}$$ (13)

$$\begin{array}{l} \min TC_{2}(t)=\sum_{a\in A}\sum_{j\in B2}\sum_{m\in M}(x_{aj}^{m}(t)\times w_{aj}^{m}(t)\times l_{a_{i}↔b_{j}}\times h)+\\ \sum_{k\in B1}\sum_{j\in B2}\sum_{m\in M}\left(y_{kj}^{m}(t)\times w_{kj}^{m}(t)\times l_{b_{k}↔b_{j}}\times h\right)+\\ \sum_{e\in E}\sum_{j\in B2}\sum_{m\in M}\left(z_{ej}^{m}(t)\times w_{ej}^{m}(t)\times l_{e_{i}↔b_{j}}\times h\right) \end{array}$$ (14)

The objective functions of the distribution model consider transportation time and cost. Eq (13) is to minimize the longest transportation time from each relief supplier to the epidemic area, Eq (14) is to minimize the total transportation cost.

There are three sets of constraints listed below.


$${\mathrm{Pr}}_{m}\left\{\begin{array}{l} \sum_{a\in A}x_{aj}^{m}(t)\times \min\left\{qa_{i}^{m}(t),w_{aj}^{m}(t)\times V_{m}\right\}+\\ \sum_{k\in N1}y_{kj}^{m}(t)\times \min\left\{qb_{k}^{m}(t),w_{kj}^{m}(t)\times V_{m}\right\}+\\ \sum_{e\in E}z_{ej}^{m}(t)\times \min\left\{qe_{i}^{m}(t),w_{ej}^{m}(t)\times V_{m}\right\}\geq D_{j}^{m}(t) \end{array}\right\}\geq \omega ,j\in N2(t)$$ (15)

$$w_{aj}^{m}(t)\leq na_{i}\;w_{kj}^{m}(t)\leq nb_{k}\;w_{ej}^{m}(t)\leq ne_{i}$$ (16)

$$na_{i},nb_{j}/nb_{k},ne_{i}\geq 0\;qa_{i}^{m}(t),qb_{j}^{m}(t)/qb_{k}^{m}(t),qe_{i}^{m}(t)\geq 0$$ (17)

$$l_{a_{i}↔b_{j}},l_{b_{k}↔b_{j}},l_{e_{i}↔b_{j}}\geq 0\;V_{m},g,h>0$$

Constraint (15) is to ensure that the amount of the medical supplies sent from the chosen suppliers can meet the demand of the epidemic area with a probably of *ω*. Particularly, the quantity of reliefs materials supplied by the epidemic areas are surplus supplies beyond their own consumption. Constraint (16) indicates the number of vehicles used to transport emergency supplies should not exceed the available number. Constraint (17) is the non-negativity constraint, which ensures that the above variables are nonnegative.

To simplify the model, this study sets an emergency rescue time threshold, and converts time constraints to time cost constraints. Specifically, if arrive time of relief supplies is beyond the scheduled time, there will be an additional time cost, otherwise the time cost is 0. The time cost is defined as the product of the penalty coefficient and delay time, the total cost consists time cost and transport cost. Furthermore, the two objective functions in Eqs (13) and (14) can be combined into a single objective function, which is to minimize the total cost. The new optimization model is given in Eq (18), it also needs to meet the constraints (15)—(17).


$$\begin{array}{l} \min TC(t)=\sum_{a\in A}\sum_{j\in B2}\sum_{m\in M}(x_{aj}^{m}(t)\times w_{aj}^{m}(t)\times l_{a_{i}↔b_{j}}\times h)+\\ \sum_{k\in B1}\sum_{j\in B2}\sum_{m\in M}\left(y_{kj}^{m}(t)\times w_{kj}^{m}(t)\times l_{b_{k}↔b_{j}}\times h\right)+\\ \sum_{e\in E}\sum_{j\in B2}\sum_{m\in M}\left(z_{ej}^{m}(t)\times w_{ej}^{m}(t)\times l_{e_{i}↔b_{j}}\times h\right)+\\ \sum_{a\in A}\sum_{j\in B2}\sum_{m\in M}\beta \times \max\left\{0,w_{aj}^{m}(t)\times l_{a_{i}↔b_{j}}\times g-T\right\}\times x_{aj}^{m}(t)+\\ \sum_{k\in B1}\sum_{j\in B2}\sum_{m\in M}\beta \times \max\left\{0,w_{kj}^{m}(t)\times l_{b_{k}↔b_{j}}\times g-T\right\}\times y_{kj}^{m}(t)+\\ \sum_{e\in E}\sum_{j\in B2}\sum_{m\in M}\beta \times \max\left\{0,w_{ej}^{m}(t)\times l_{e_{i}↔b_{j}}\times g-T\right\}\times z_{ej}^{m}(t) \end{array}$$ (18)

## Solution algorithm

**Fig 4:**
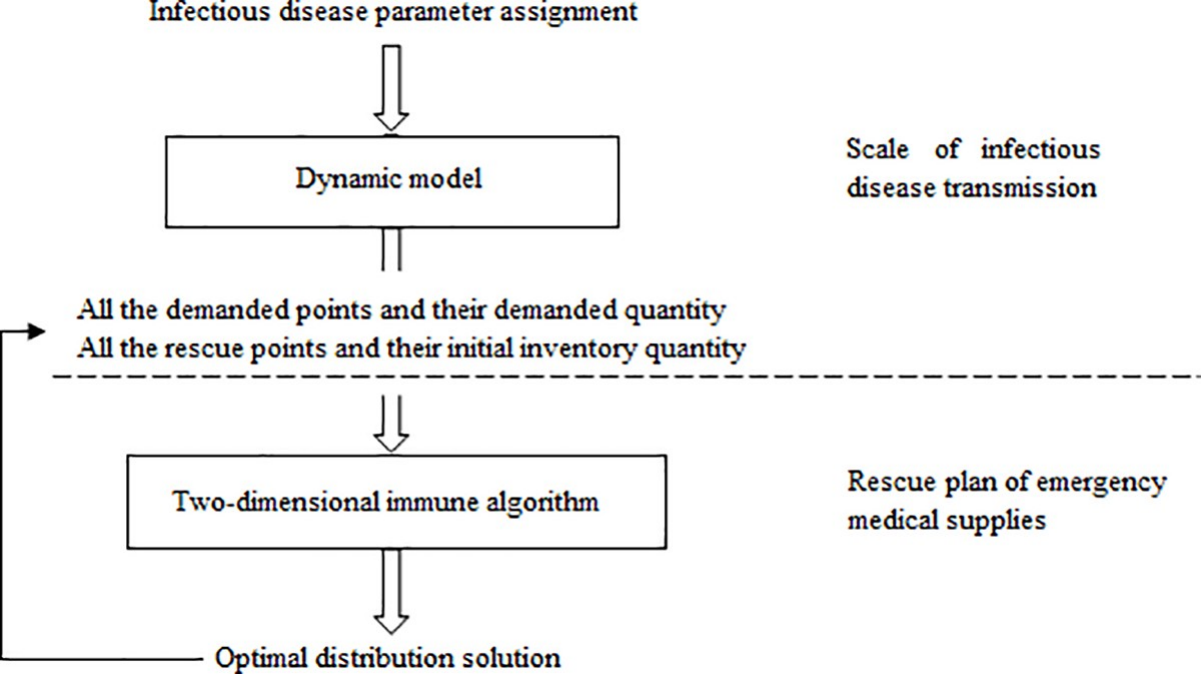
Model solution idea.

This section introduces the solving algorithm of the model. As shown in Fig 4, The solving algorithm is based on a two-step process. The modified SEIAR model and relief supplies demand forecast can be calculated by Eqs (6)—(10) and Eqs (11)—(12) respectively. Combined with the quantity of emergency medical supplies in each region, the epidemic areas that can be used as rescue points, and the epidemic areas that need to be rescued can be determined. As well as the quantity of different relief materials that could be distributed at each rescue point and the amount that be demanded by each demand points. The distribution decision model is a multi-objective stochastic programming problem with multiple rescue points, multiple demand points and a variety of relief materials. Therefore, this paper uses the two-dimensional immune algorithm to solve the distribution problem. The algorithm operation instructions as follow.

### Antibody coding mode

**Fig 5:**
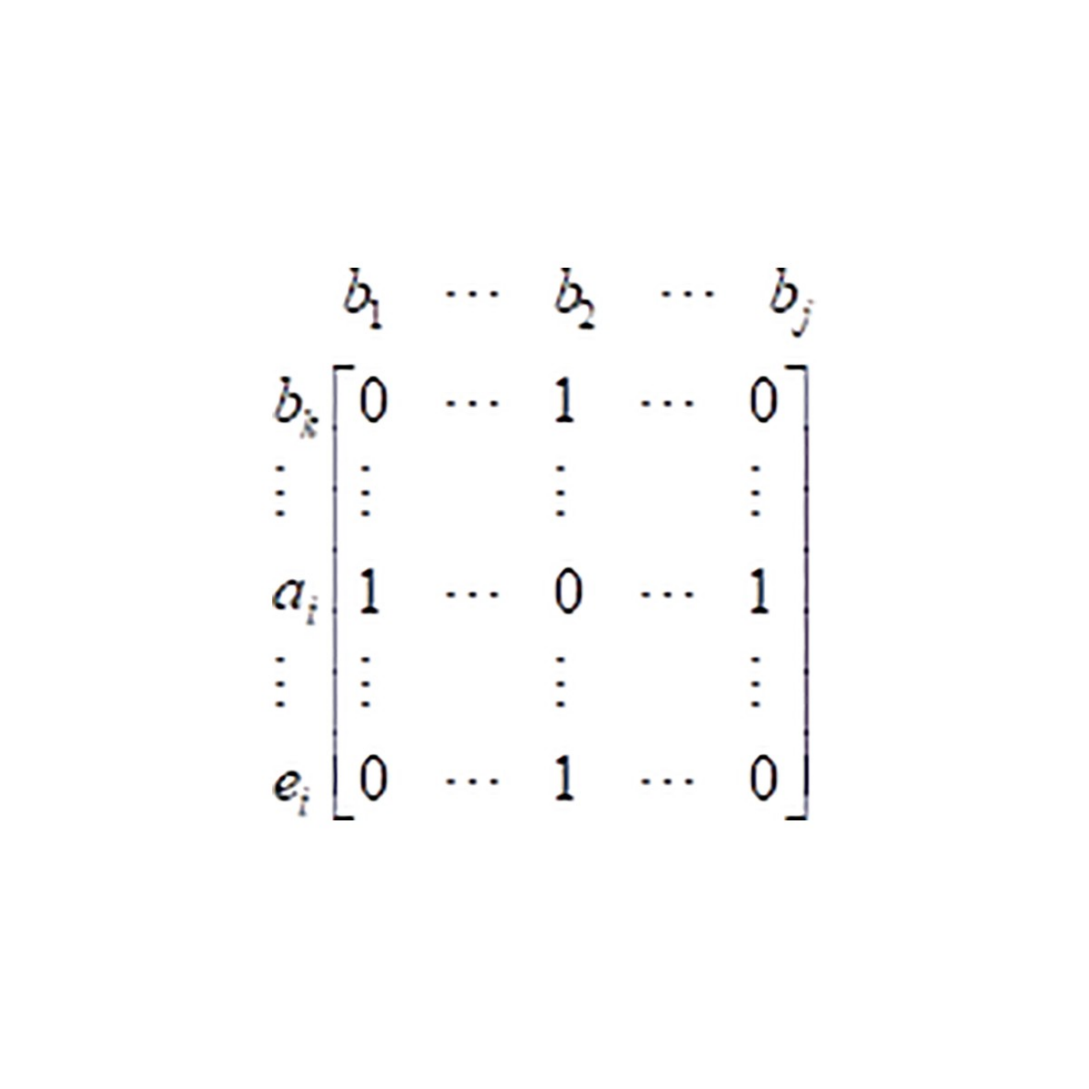
Two-dimensional binary antibody coding.

Considering the distribution decision model is a two-dimensional combinatorial optimization problem, this paper uses two-dimensional binary coding mode. As shown in Fig 5, each column of the antibody represents a demand point *b*_*j*_, each row represents a rescue point *b*_*k*_, *a*_*i*_ or *e*_*i*_, and the genes of antibodies is $x_{ai}^{m}(t)$, $y_{kj}^{m}(t)$, $z_{ej}^{m}(t)$.

However, Fig 5 can only show which rescue point was involved in the rescue, but the quantity of allocated supplies cannot be determined. In the process of distribution, there will be some problems such as the total amount of relief supplies at all rescue points cannot meet the demand of a demand point, or the total amount of relief supplies allocated by a rescue point exceeds its own reserve, so that the antibody becomes an illegal code. In order to solve these problems, the genetic code needs to be revised. In the revision strategy, it must be clear that the actual contribution amount of relief supplies of each rescue point *b*_*k*_, *a*_*i*_ or *e*_*i*_, to the demand point *b*_*j*_, and the remaining amount of relief supplies of *b*_*k*_, *a*_*i*_ or *e*_*i*_. Once all the emergency relief supplies of *b*_*k*_, *a*_*i*_ or *e*_*i*_ have been contributed, it will no longer respond to the emergency requests of other demand points. Specific revision steps can be referred to reference [10].

### Fitness function

The set of the population is *E*, *E* = {*e*_1_,*e*_2_,⋯,*e*_*s*_}. The population size is *S*, *e*_*s*_(*s*∈{1,2,⋯,*S*}) is the *s*-th antibody, and the fitness function is given in Eq (19). *Z*_*s*_ is the objective function value of antibody *E*, and 0≤*Num*≤*j* is the total number of demand points to be satisfied.


$$f\left(e_{s}\right)=Z_{s}+\frac{1}{Num}$$ (19)

Obviously, the value of *f*(*e*_*s*_) is inversely proportional to *Num* and directly proportional to *Z*_*s*_. This means that the *f*(*e*_*s*_) value will be larger according to the transportation cost and response time are reduced, as well as more demand points are satisfied.

### Crossover and mutation operators

**Fig 6:**
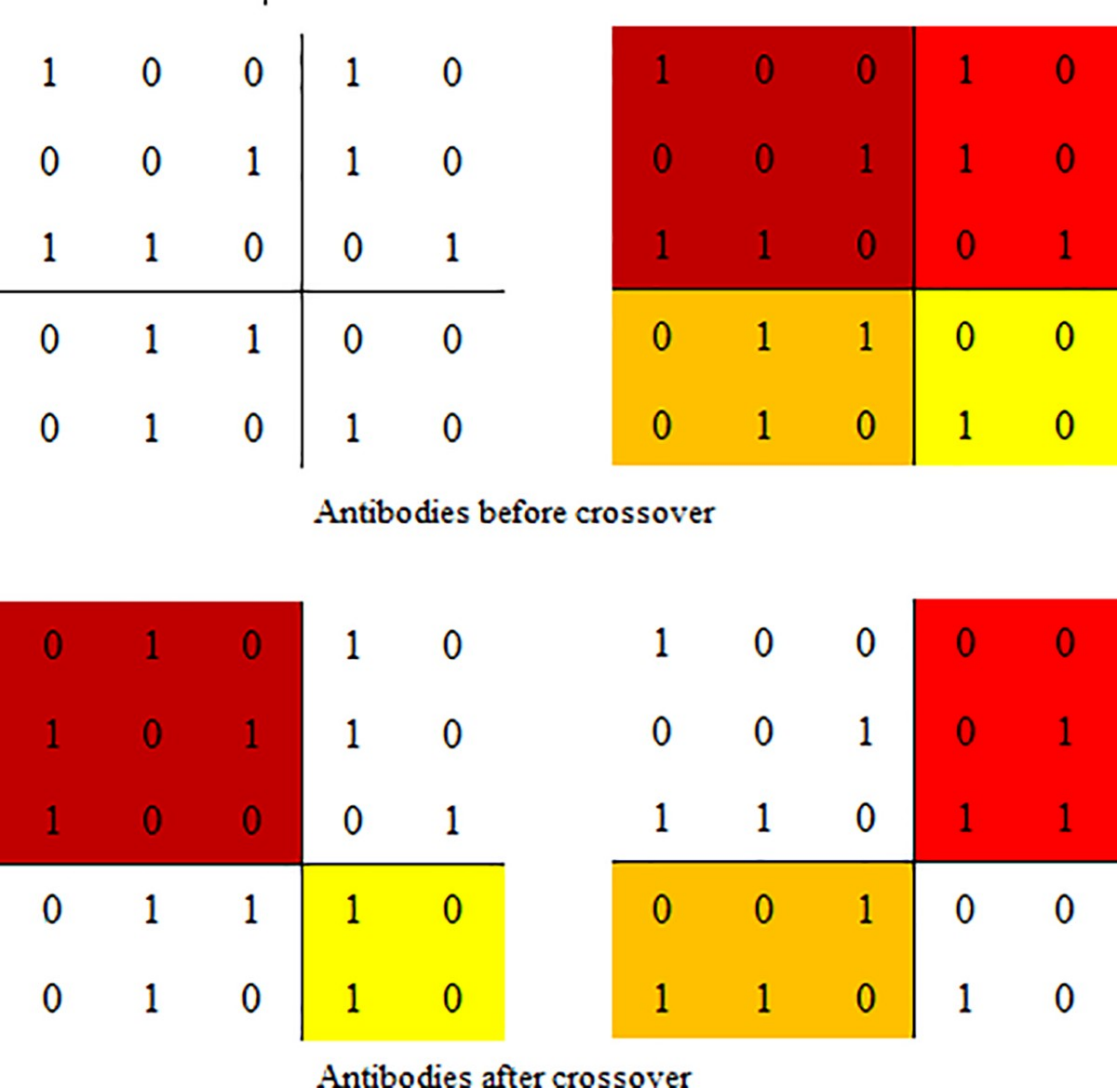
Random block crossover operation.

**Fig 7:**
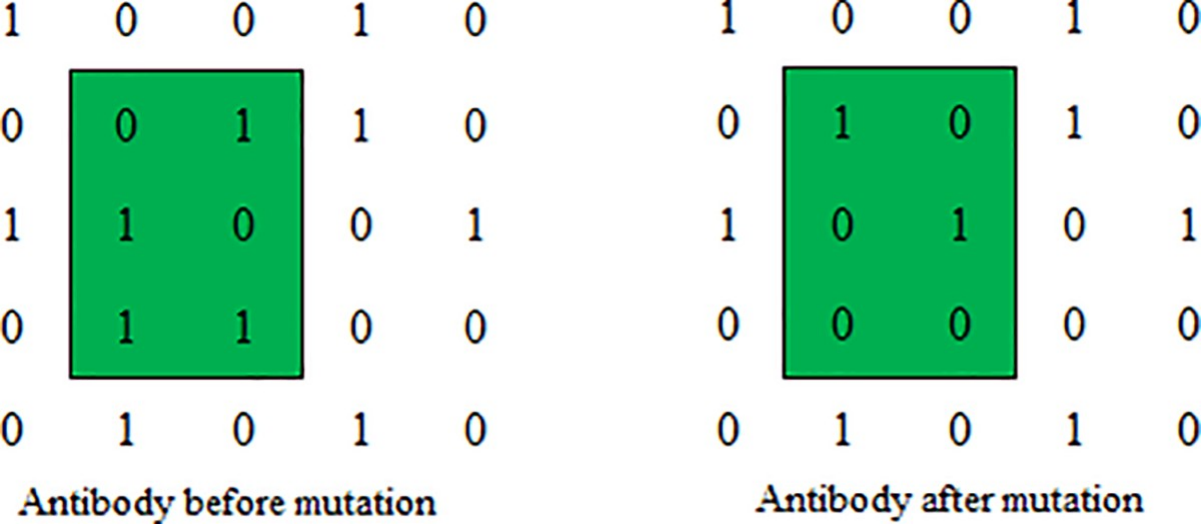
Random block mutation operation.

In this paper, random block crossover and random block mutation were used for antibody crossover and mutation respectively. As shown in Fig 6, The antibody is divided into four regions by a random gene site which is selected from the antibody, and different regions are combined to produce different new antibodies. As shown in Fig 7, A random region is constructed by two random gene sites which are selected from the antibody, and all the values in the region are swaped by 0↔1.

### Vaccine distill

The automatic vaccine extraction method proposed in reference [30] was adopted in this paper. Several excellent individuals were selected from each generation of antibodies to form the memory bank, and then the vaccine was extracted from the updated memory bank.

### Immune operator

**Fig 8:**
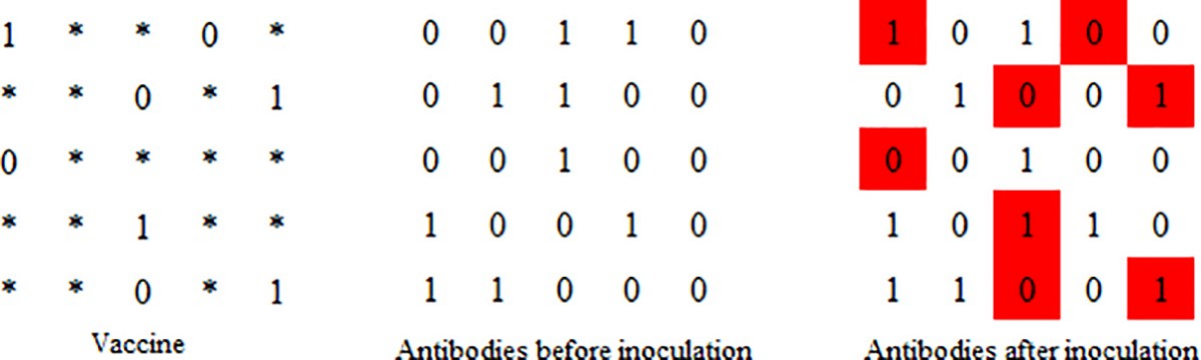
Vaccination operation.

The immune operator includes vaccination and immunoselection. Vaccination is the modification of certain gene sites of the antibody(as shown in Fig 8), so that the modified antibody has a higher fitness value in a greater probability. Immunoselection refers to the detection of new antibodies generated by vaccination. If the fitness of the new antibody is lower than the original antibody, the original antibody will join in the new generation population. Otherwise, the new antibody will join in the new generation population.

### Steps of algorithm

**Fig 9:**
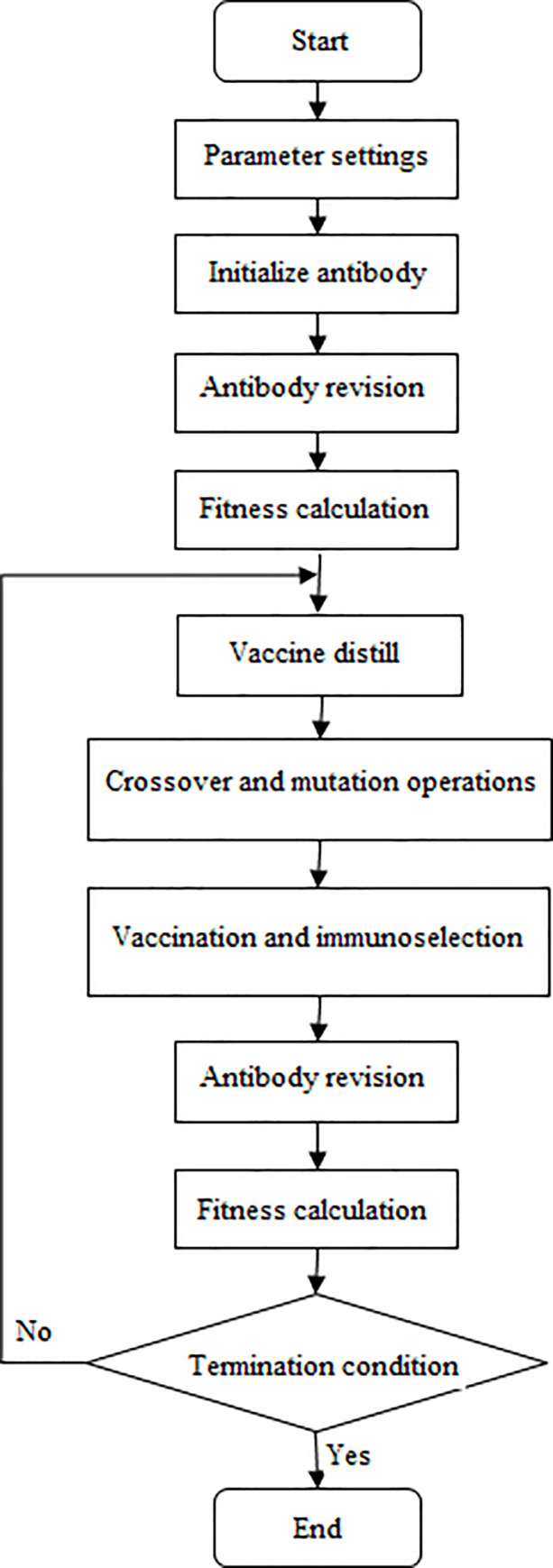
Flow chart of two-dimensional immune algorithm.

The calculation process of the immune algorithm is shown in Fig 9. Parameter settings include antibody population size, maximum iterations, crossover probability and mutation probability. Specially, the new antibody generated by the crossover and mutation operations may become illegal code, so the new antibody needs to be revised again to ensure its legality.

## Numerical study

Numerical studies with the COVID-19 occurring in Hubei province, China are conducted, and the corresponding results indicate the applicability of the proposed method and its potential advantages. All computational processes are conducted with MATLAB on a personal computer with a 2.70 GHz CPU and 4G RAM. Following an introduction of the case background, the main procedures executed in this numerical study are the validation of forecasting model, and experimental testing.

### Background

The COVID-19 outbreak originated in Wuhan city of Hubei Province, China, in December 2019. The epidemic spread across the entire country quickly. When the severity of this public health emergency was determined, the Chinese central government responded. In order to prevent the spread of the epidemic, the government locked down the Wuhan city on January 23, 2020.

The numerical study focuses on Hubei province, which is located on the center of China. Hubei Province has 17 cities, of which Wuhan is the capital. Wuhan is one of the largest transit hubs of china, which has transported more than 15 million passengers per year during the Spring Festival travel rush in recent years. Wuhan registers 10 million permanent residents, as well as 5 million migrants who lived in the city for at least six months of the year. Particularly, almost 70% of the migrants living in Wuhan come from other cities of Hubei province. Given the epidemic-related information and the basic state of the logistics network, the proposed methodology is used to forecast the spread trend of the COVID-19 and to make medical logistics decisions for Hubei province. The unit interval is one day.

**Fig 10:**
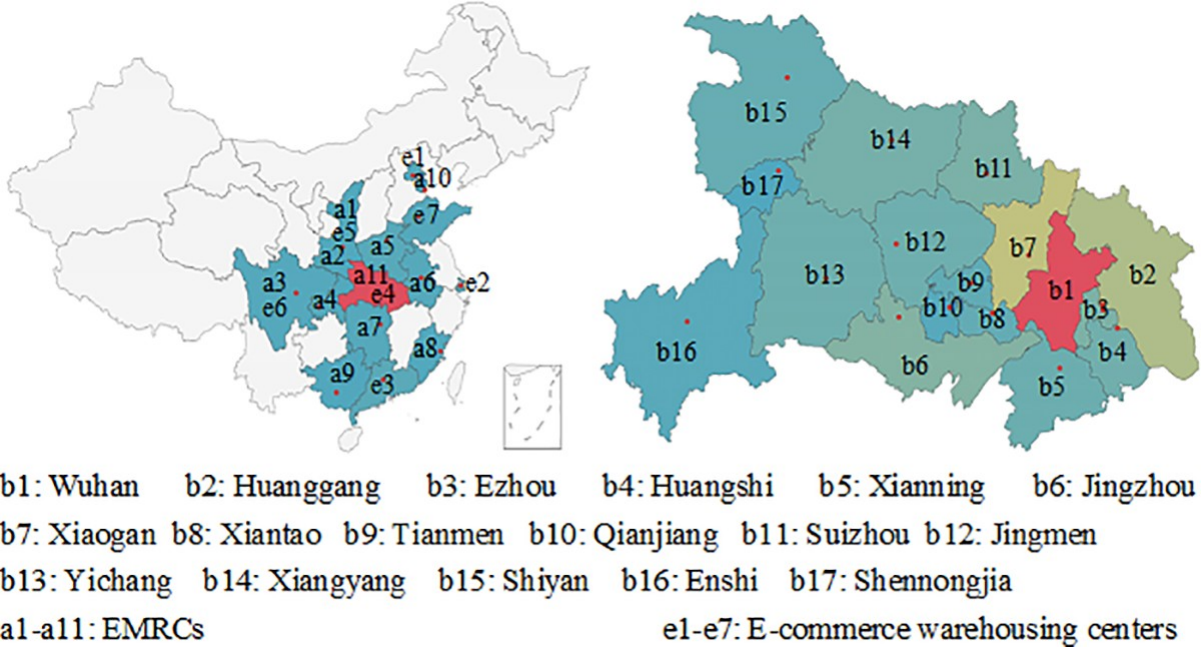
Study areas.

The COVID-19 spread quickly across the entire province from Wuhan. The related cities include Wuhan, Huanggang, Ezhou, Huangshi, Xianning, Jingzhou, Xiaogan, Xiantao, Tianmen, Qianjiang, Suizhou, Jingmen, Yichang, Xiangyang, Shiyan, Enshi, and Shennongjia. There are 11 national EMRCs and 7 E-commerce warehousing centers can send relief supplies to Hubei province. Fig 10 shows the simplified geographical relationships among these affected areas, national EMRCs and E-commerce warehousing centers.

### Testing of the forecasting model

This study compares the numbers of corresponding demanders instead of comparing the quantity of demands, because demands for testing supplies are positively correlated with the numbers of exposed individuals, and demands for treatment supplies are positively correlated with the numbers of infectious individuals, as well as demands for prophylactic supplies are correlated with the number of exposed, infectious, and asymptomatic people. The forecasting numbers of exposed, infectious and asymptomatic individuals are given according to Eqs (1)–(5) and Eqs (6)–(10).

The parameters are presented in S1 Appendix, and they are set as follows: (1) the parameters for population (permanent people, natural mortality rate, mobility rate) are set (Hubei Provincial Bureau of Statistics, 2019; Baidu Map Spring Festival migration big data, 2020). (2) The parameters for disease (transmission rate, asymptom rate, recovery rate, disease mortality rate and incubation period) are set. The value of these disease parameters can shortly be estimated by doctors and medical experts after a new disease appeared in a region. In this work, they are obtained from several medical reports and statistics (World Health Organization, 2020; Chinese Center for Disease Control and Prevention). (3) Infection rates are set with two methods. First, the rate can be obtained by fitting the forecasting curve with observations, on the premise of adequate infectious cases in the epidemic areas. In this work, the infection rate in Wuhan (j = 1) is set in this manner. In epidemic areas with a few infectious cases, these rates can be determined by comparing specific areas with previous cases of similar epidemic outbreak. In this study, infection rates in the other cities (j = 2–17) are set in this manner.

In order to indicate the applicability of the modified SEIAR model, this work compares the proposed model with standard SEIAR model and the observed data. The following figure compares demand forecasting in Wuhan (j = 1) as an example. The results of other cities (j = 2–17) are listed in S2 Appendix.

**Fig 11:**
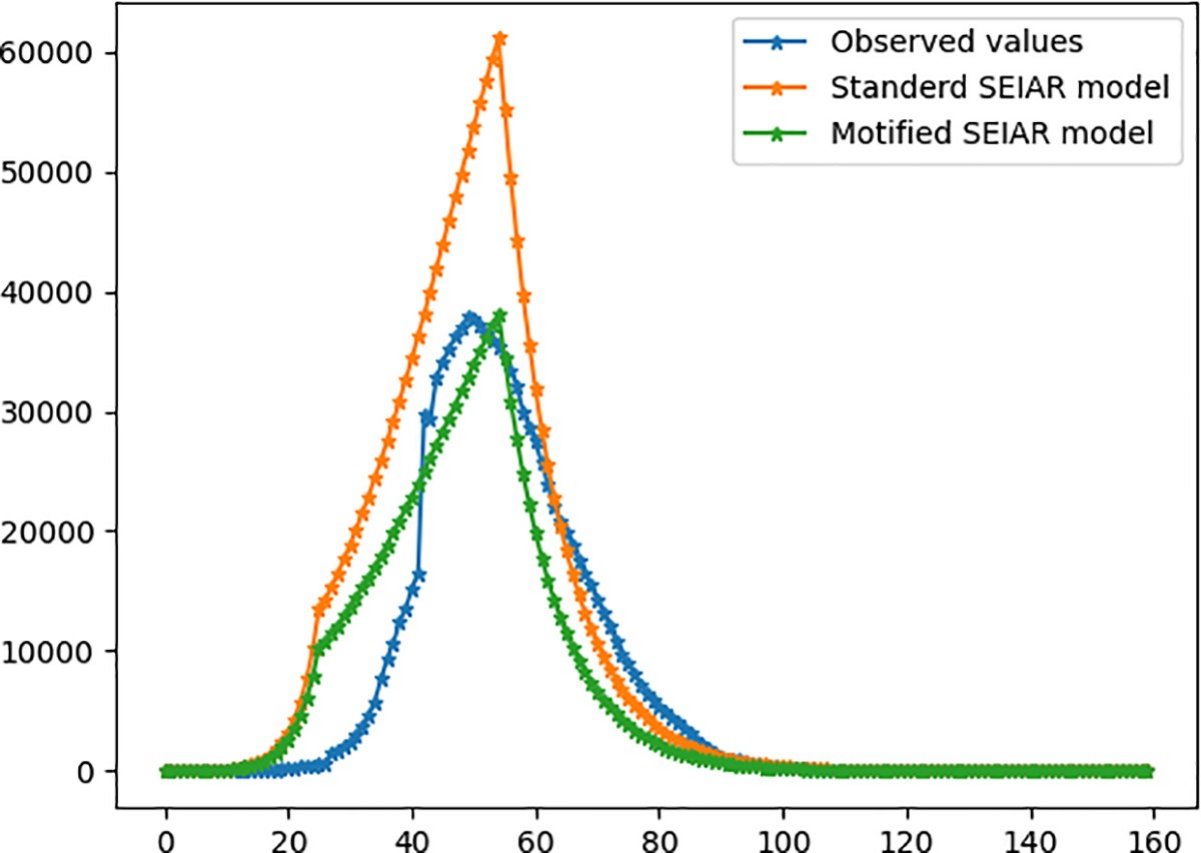
Forecasting of treatment demanders.

Fig 11 shows that the modified method proposed in this paper perform better than standard SEIAR model, the forecasting values of which are closer to the actual values. However, the forecasting values of both models are larger than the actual value before the peak and slightly smaller than the actual value after the peak. The main reason for this trend is the hysteresis in the recognition, identification, and diagnosis of new infectious diseases in the initial stage, the absence of specific medicine for COVID-19 until now, and the occasional re-positive phenomenon. Nonetheless, this forecasting accuracy is enough to facilitate distribution decision-making.

### Testing of the distribution model

**Fig 12:**
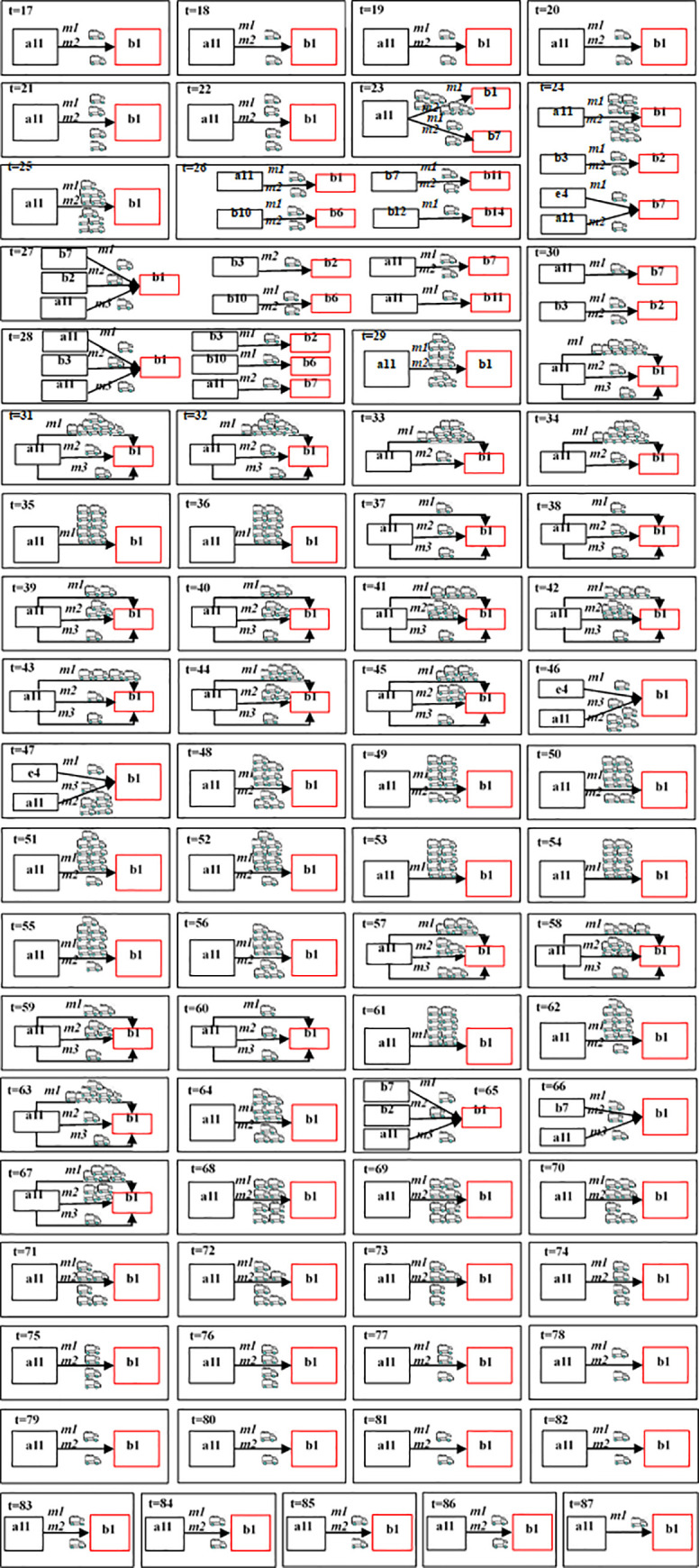
Emergency medical supplies distribution decisions of the proposed method.

**Fig 13:**
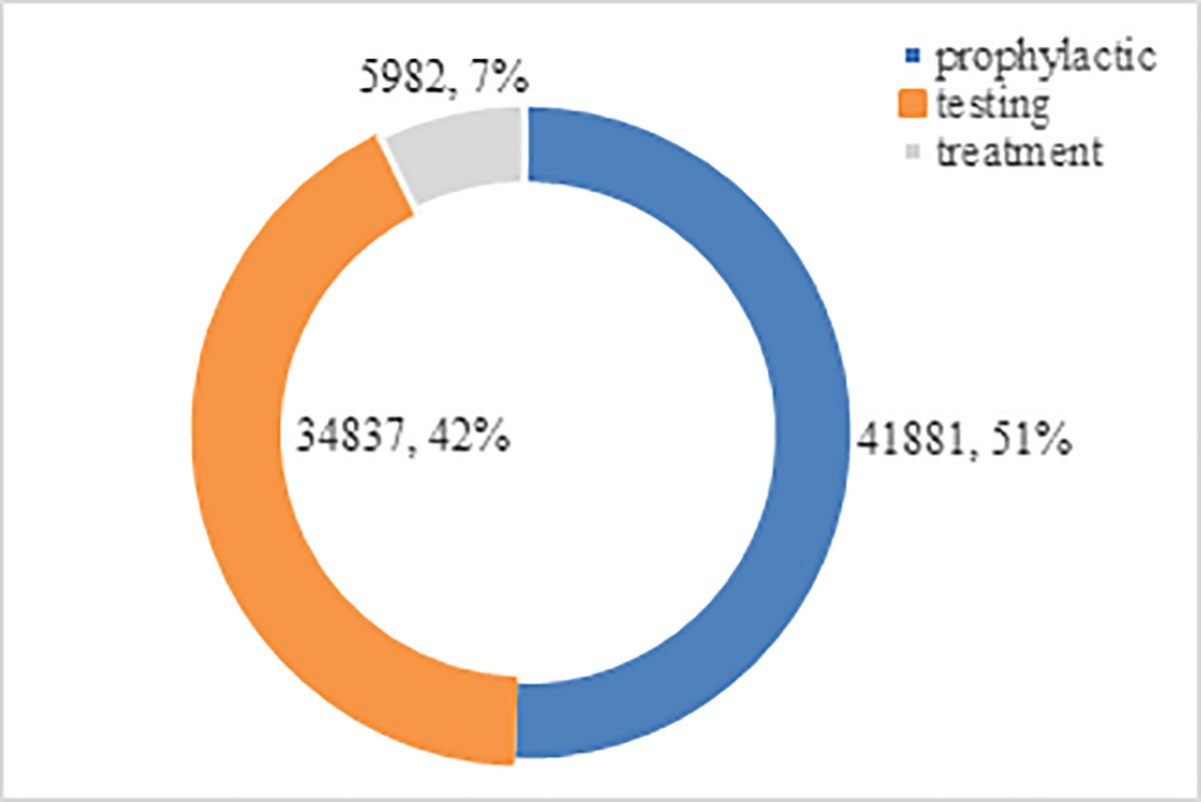
Distribution cost of the proposed method.

In this section, the proposed distribution method is tested. The parameters and statistics are reported in S1 Appendix, and the results are listed in Figs 12 and 13.

Fig 12 shows the emergency medical supplies distribution decisions of the proposed method. The emergency supplies distribution began in 17th period, and finished in 87th period. Wuhan(b1) is the most affected area by the epidemic, and the demand for emergency medical supplies are greatest, the demanded supplies of which are mainly sent from the national EMRC in Wuhan(a11), as well as from the E-commerce warehousing centers in Wuhan(e4), and the neighboring epidemic areas (b2, b3, b7).

The total cost of the distribution decisions is 82700. As shown in Fig 13, allocation cost of prophylactic materials is the largest, according to the most numerous demanders. Distribution cost of testing reliefs is slightly lower than prophylactic, due to the demand of testing supplies needed by per demander is more than other supplies, although the number of demanders is much less than prophylactic supplies.

## Discussion

In this section, four scenarios are designed, and an analysis is conducted to compare these observations in different scenarios.

We test four typical types of scenarios as follows. Scenario 1: traditional distribution system without considering epidemic areas and e-commerce warehousing centers as the rescue points; Scenario 2: distribution system without considering epidemic areas as the rescue points; Scenario 3: distribution system without considering e-commerce warehousing centers as the rescue points; Scenario 4: the proposed method. The varying parameters are listed in Table 1.

**Table 1:** Parameter adjustment of different scenarios.

Scenario	Description	Adjustment
1	Minimize the available amount of relief supplies in e-commerce warehousing center, and maximize the distance between different epidemic areas.	$qe_{i}^{m}(t)=0$, $l_{b_{k}↔b_{j}}=\infty$
2	Maximize the distance between different epidemic areas.	$l_{b_{k}↔b_{j}}=\infty$
3	Minimize the available amount of relief supplies in e-commerce warehousing center.	$qe_{i}^{m}(t)=0$
4	The proposed method.	------

The other parameters are same to those in *Testing of the distribution model*. We make distribution decisions in the three situations as in the analysis with *Testing of the distribution model*.

**Fig 14:**
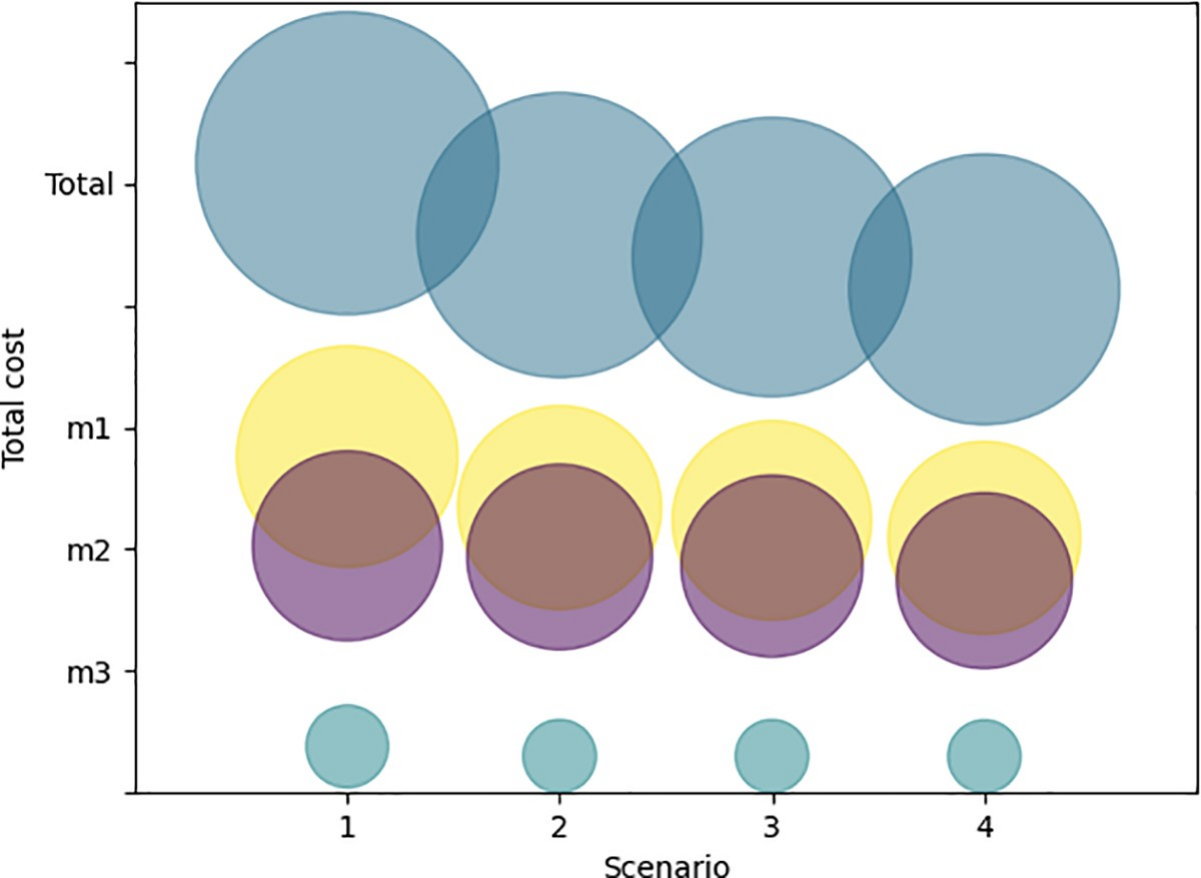
Cost comparison of the four scenarios.

The objective function values of the three scenarios are presented in Table 2. The comparison of the four scenarios is presented in Fig 14.

**Table 2:** Objective function values of different scenarios. unit: RMB.

Scenario	Objective function values (cost)			
	prophylactic	testing	treatment	total
1	55222	40559	7620	103401
2	46842	38731	6018	91591
3	44735	37241	6012	87988
4	41881	34837	5982	82700

The following observations are made based on the results of experimental testing:

1. The proposed method (Scenario 4) is optimal. The total cost is 6% lower than without considering cooperation of different epidemic areas (Scenario 2), and 9.7% lower than without considering cooperation of e-commerce warehousing centers (Scenario 3). Particularly, the total cost is 20% lower than without considering any cooperation (Scenario 1).
2. The costs of Scenario 2–4 are not significantly different, because of the most severely affected area is Wuhan, there is a national EMRC, a large e-commerce warehousing center and a local EMRC in the city, therefore the supplies distribution is mainly transported within the Wuhan city in most period.
3. The cost of Scenario 3 is lower than Scenario 2, due to the distribution decision in other epidemic areas near Wuhan. Without considering cooperation of different epidemic areas, the demands of epidemic areas b2-b17 will be transported from Wuhan city, which would increase the distribution cost.
4. In the rescue of multi-epidemic areas, if the epidemic areas carry out mutual rescue, it will greatly reduce the transportation cost. If non-governmental organizations such as e-commerce warehousing centers are mobilized, relief will be more effective.

## Conclusions

This paper presents a method for emergency medical logistics to quick response to emergency epidemics. The proposed methodology includes two recursive mechanisms: (1) the time-varying forecasting of medical supplies demand according to a modified SEIAR epidemic diffusion model, (2) relief distribution base on a Multi-objective dynamic stochastic programming model. Specially, the distribution model addresses a hypothetical network of emergency medical logistics with considering national EMRCs, other epidemic areas and e-commerce warehousing centers as the rescue points.

A numerical study conducted on a real COVID-19 outbreak in Hubei province of China, the results show the applicability of the proposed method. Three experimental situations are tested to support and supplement the analysis with real data, and the testing results demonstrate the advantages of the proposed model. Further, according to the findings of the scenarios, managerial insights are provided.

Based on the proposed method, the performance of emergency medical logistics may be improved significantly. The first mechanism of the proposed model is used in demand forecast, and base on the forecast results, the appropriate supplies dispatching mechanism can be chosen. Furthermore, an emergency medical logistics network is applied to distribute limited medical reliefs to multiple epidemic areas, of which the suppliers (rescue points) and the demanders (demand points) are dynamic through the rescue periods. In addition, considering the mutual rescue between epidemic areas, the increasingly mature development of e-commerce, emergency medical materials are supplied by the national EMRCs, as well as by the less affected area of all nearby epidemic areas and the e-commerce warehousing centers.

Academic literature indicate that relief supplies are one of the critical factors to emergency rescue, and the medical supplies are mainly delivered from national and local EMRCs. This paper focuses on the cooperation of nearby epidemic areas with different degrees of contagion, as well as the cooperation of government and non-governmental organizations, by considering the epidemic areas and e-commerce warehousing centers as one of the rescue points. This work highlights the necessity and feasibility of promoting cooperation between different participants in a public emergency. The government plays the dominant role in emergency rescue, but non-governmental organizations is equally important in providing or donating relief supplies. The local governments are independent, but they can strengthen cooperation in emergency rescue to contain the epidemic as soon as possible, according to the rapidly spread of the epidemic. Numerical results show that with cooperation of different sectors, the relief can be more effective. In this study, the proposed emergency logistics network includes three various type of rescue point, focuses on the optimization of transportation cost, but without considering the cooperation mechanisms and cooperation cost between government and electronic commercial enterprise. In addition, pharmaceutical companies, an important provider of medical supplies, should also be involved in the emergency medical logistics network. Therefore, the cooperation mechanism between government and non-governmental organizations in emergency rescue, and how to set up the emergency medical supplies dispatching network under the cooperation mechanism are worthy of future research. Such research is particularly important for medical logistics and rescue in large public health emergencies. For the multi-objective dynamic stochastic programming model established in this paper, two-dimensional immune algorithm is adopted to solve the problem, but the computation time needs to be reduced and the computational efficiency needs to be improved. Therefore, more research is necessary to the algorithm innovation.

We hope that this study can not only provide the government with a new idea of dispatching emergency relief supplies, that the rescue efficiency can be improved by mutual rescue between epidemic areas, but also stimulate more excellent researches taking attention to the cooperative of all participants in emergency rescue.

## Supporting information

S1 Appendix Values of parameters in the numerical study. There are four tables, including parameters for epidemic and population, mobility rate, medical reliefs, and other parameters.
(PDF)

S2 Appendix There is a figure named “comparison in forecasting and observed values of infected numbers in other cities”. (PDF)
