# Supplementary material for: Methodology of emergency medical logistics for multiple epidemic areas in public health emergency
Source: PLoS One. 2021 Jul 26;16(7):e0253978. doi: 10.1371/journal.pone.0253978 (PMC8312947; doi:10.1371/journal.pone.0253978)
Supplement: S1 Appendix — There are four tables, including parameters for epidemic and population, mobility rate, medical reliefs, and other parameters. (PDF) [file pone.0253978.s001.pdf]

S1 Appendix

The values of parameters in the numerical study are listed in S1 Tables1-4.

S1 Table 1. Parameters for epidemic and population.

Area	N_j	S_j (0)	E_j (0)	I_j (0)	A_j (0)	R_j (0)	λ_j^1	λ_j^2	λ_j^3	σ_j	β_j(‰)	γ_j	α_j(‰)	d_j(‰)
j = 1	11212000	11211973	0	27	0	0	6.4	2.15	0	0.097	1.6	0.93	3.7	5.7
j = 2	6333000	6333000	0	0	0	0	12.03	0	0	0.097	1.6	0.95	5	4.98
j = 3	1077700	1077700	0	0	0	0	12.95	0	0	0.097	1.6	0.95	5	5.86
j = 4	2471700	2471700	0	0	0	0	12.5	0	0	0.097	1.6	0.93	4.7	6.23
j = 5	2548400	2548400	0	0	0	0	11.45	0	0	0.097	1.6	0.95	2.5	4.62
j = 6	5570100	5570100	0	0	0	0	12.57	0	0	0.097	1.6	0.95	4	6.51
j = 7	4921000	4921000	0	0	0	0	12.12	0	0	0.097	1.6	0.93	3.9	4.58
j = 8	1140100	1140100	0	0	0	0	12.2	0	0	0.097	1.6	0.92	4.5	8.34
j = 9	1272300	1272300	0	0	0	0	12.9	0	0	0.097	1.6	0.95	4	7.08
j = 10	966000	966000	0	0	0	0	12	0	0	0.097	1.6	0.95	6	6.27
j = 11	2221000	2221000	0	0	0	0	13.9	0	0	0.097	1.6	0.93	3.8	7.08
j = 12	2896300	2896300	0	0	0	0	12.7	0	0	0.097	1.6	0.93	5	5.5
j = 13	4137900	4137900	0	0	0	0	13.4	0	0	0.097	1.6	0.93	4.3	6.93
j = 14	5680000	5680000	0	0	0	0	13.25	0	0	0.097	1.6	0.93	3.6	7.08
j = 15	3398000	3398000	0	0	0	0	13.5	0	0	0.097	1.6	0.95	2	7
j = 16	3390000	3390000	0	0	0	0	11.4	0	0	0.097	1.6	0.95	4.2	6.19
j = 17	76700	76700	0	0	0	0	13	0	0	0.097	1.6	0.95	0	9

The value of transmission rate varies in different stages according to the epidemic prevention measures taken by local governments. Wuhan (j = 1) government reported COVID-19 cases for the first time on December 31, 2019, locked down the city on January 23, 2020, and taken measure to leave no one unattended in the critical stage of epidemic control on February 21. Therefore, the transmission rate of Wuhan is divided into three stages. Other cities (j = 2-17) were locked down on January 23 or 24, 2020, and the transmission rate is divided into two stages.

S1 Table 2. Parameters for mobility rate b_{kj} and b_{jk} (t = 1-24).

Area	1	2	3	4	5	6	7	8	9	10	11	12	13	14	15	16	17
1	0	5.96	1.89	1.75	2.35	2.97	6.22	1.35	0.95	0.53	1.43	1.47	1.29	1.80	0.85	0.85	0.02
2	0	0	0	0	0	0	0	0	0	0	0	0	0	0	0	0	0
3	0	0	0	0	0	0	0	0	0	0	0	0	0	0	0	0	0
4	0	0	0	0	0	0	0	0	0	0	0	0	0	0	0	0	0
5	0	0	0	0	0	0	0	0	0	0	0	0	0	0	0	0	0
6	0	0	0	0	0	0	0	0	0	0	0	0	0	0	0	0	0
7	0	0	0	0	0	0	0	0	0	0	0	0	0	0	0	0	0
8	0	0	0	0	0	0	0	0	0	0	0	0	0	0	0	0	0
9	0	0	0	0	0	0	0	0	0	0	0	0	0	0	0	0	0
10	0	0	0	0	0	0	0	0	0	0	0	0	0	0	0	0	0
11	0	0	0	0	0	0	0	0	0	0	0	0	0	0	0	0	0
12	0	0	0	0	0	0	0	0	0	0	0	0	0	0	0	0	0
13	0	0	0	0	0	0	0	0	0	0	0	0	0	0	0	0	0
14	0	0	0	0	0	0	0	0	0	0	0	0	0	0	0	0	0
15	0	0	0	0	0	0	0	0	0	0	0	0	0	0	0	0	0
16	0	0	0	0	0	0	0	0	0	0	0	0	0	0	0	0	0
17	0	0	0	0	0	0	0	0	0	0	0	0	0	0	0	0	0

The units of the mobility rate are one in ten thousand. The COVID-19 outbreak originated in Wuhan, and spread to other cities, without backward propagation in the initial stage. Therefore, in order to simplify the calculation, only the number of people moving from Wuhan to other cities is considered, while the migration from other cities to Wuhan and the population movement between other cities are ignored. According to the lockdown measure on January 23, 2020, this parameter was set to 0 after the 24th day.

S1 Table 3. Parameters for medical reliefs.

Area	Supplies storage			number of vehicles	Area	Supplies storage			number of vehicles
	m1	m2	m3			m1	m2	m3	
b1	80000	50000	30000	5	a2	100000	80000	50000	10
b2	30000	20000	10000	5	a3	100000	80000	50000	10
b3	30000	20000	10000	5	a4	100000	80000	50000	10
b4	30000	20000	10000	5	a5	100000	80000	50000	10
b5	30000	20000	10000	5	a6	100000	80000	50000	10
b6	30000	20000	10000	5	a7	100000	80000	50000	10
b7	30000	20000	10000	5	a8	100000	80000	50000	10
b8	30000	20000	10000	5	a9	100000	80000	50000	10
b9	30000	20000	10000	5	a10	100000	80000	50000	10
b10	30000	20000	10000	5	a11	100000	80000	50000	10
b11	30000	20000	10000	5	e1	100000	0	50000	30

b12	30000	20000	10000	5	e2	100000	0	50000	30
b13	30000	20000	10000	5	e3	100000	0	50000	30
b14	30000	20000	10000	5	e4	100000	0	50000	30
b15	30000	20000	10000	5	e5	100000	0	50000	30
b16	30000	20000	10000	5	e6	100000	0	50000	30
b17	30000	20000	10000	5	e7	100000	0	50000	30
a1	100000	80000	50000	10					

S1 Table 4. Other parameters.

name	value	name	value	name	value	name	value
a^{m1}	1	L	4	v	8000	β	100
a^{m2}	2	g	90	T_w	1		
a^{m3}	1	h	3	w	0.95		
