# Supplementary material for: Methodology of emergency medical logistics for multiple epidemic areas in public health emergency
Source: PLoS One. 2021 Jul 26;16(7):e0253978. doi: 10.1371/journal.pone.0253978 (PMC8312947; doi:10.1371/journal.pone.0253978)
Supplement: S2 Appendix — (PDF) [file pone.0253978.s002.pdf]

S2 Appendix

The comparison in forecasting and observed values of infected numbers in other cities (j = 2-17) is listed in the figure below.

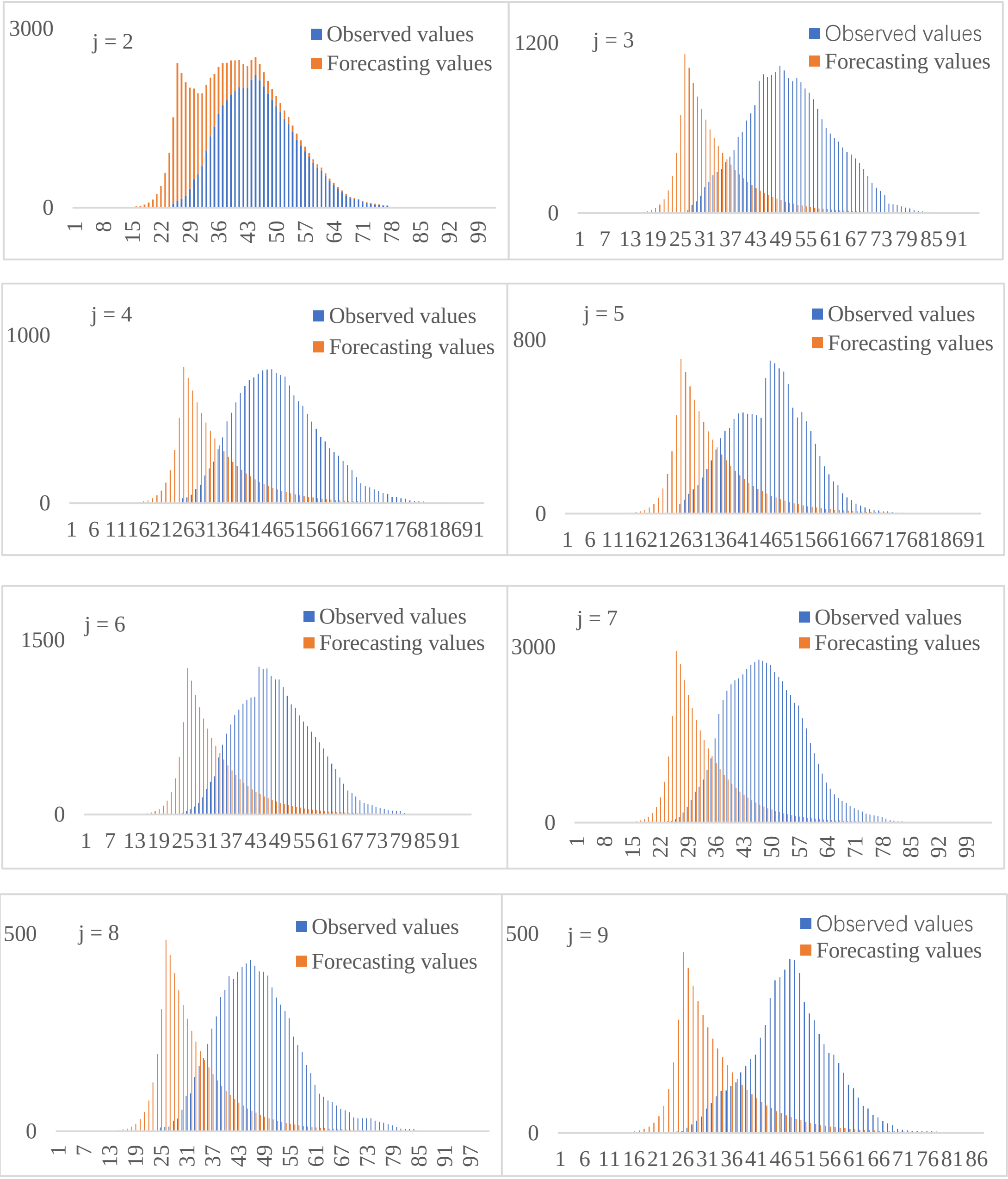

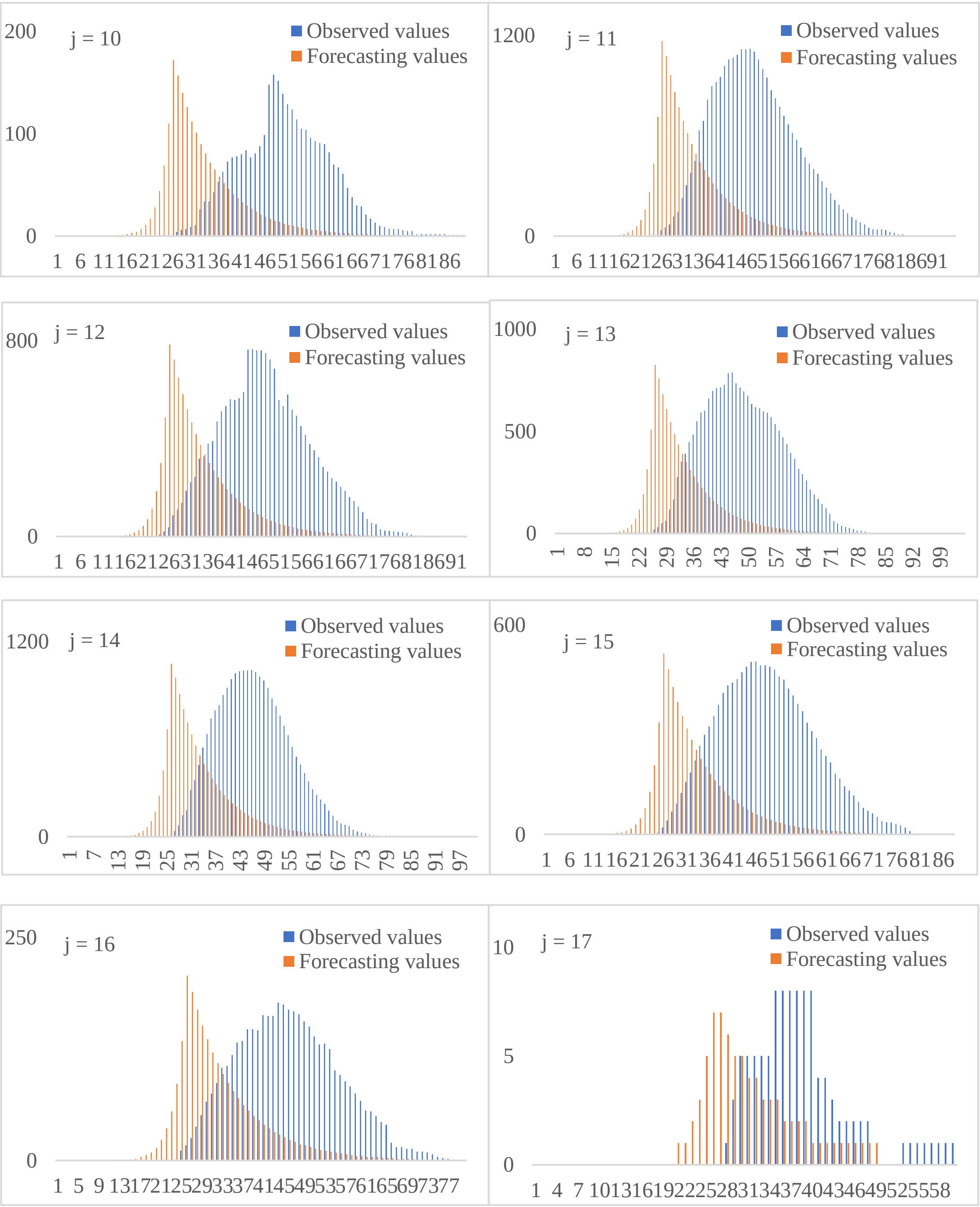

Fig. Comparison in forecasting and observed values of infected numbers in other cities
